# The p75^NTR^ and its carboxyl‐terminal fragment exert opposing effects on melanoma cell proliferation and apoptosis via modulation of the NF‐κB pathway

**DOI:** 10.1002/2211-5463.13047

**Published:** 2020-12-16

**Authors:** Maojiao Zhong, Yingying Wang, Farrukh Nisar Muhammad, Jing Gao, Chunxiang Bian

**Affiliations:** ^1^ Key Laboratory for Molecular Biology and Biopharmaceutics School of Life Science and Technology Mianyang Normal University China; ^2^ Department of Endodontics Stomatological Hospital of Chongqing Medical University China; ^3^ Department of Stomatology Daping Hospital Army Medical University (Third Military Medical University) Chongqing China; ^4^ Department of Physiology and Biochemistry Cholistan University of Veterinary and Animal Sciences (CUVAS) Bahawalpur Pakistan

**Keywords:** apoptosis, NF‐κB, p75^NTR^, p75^NTR^‐CTF, sorafenib

## Abstract

The p75 neurotrophin receptor (p75^NTR^), a member of the tumor necrosis factor superfamily of receptors, is sensitive to proteolysis and has been observed to be expressed in various cancers. However, the roles of p75^NTR^ and its proteolytic fragments in tumorigenesis remain incompletely understood. Here, we report that the proportion of the p75^NTR^ carboxyl‐terminal fragment (p75^NTR^‐CTF) is much higher than that of the full‐length p75^NTR^ (p75^NTR^‐FL) in melanoma cells. Whereas p75^NTR^‐FL positively regulates apoptosis, p75^NTR^‐CTF promotes cell proliferation and survival, as well as increasing sorafenib resistance *in vivo* and *in vitro*. Moreover, p75^NTR^‐CTF activates the nuclear factor kappa B pathway and enhances the mRNA and protein levels of its downstream genes *c‐IAP1/2*, *FLIP*, *bFGF*, *IL8* and *VEGF*. On the contrary, p75^NTR^‐FL inhibits these processes. Taken together, these findings demonstrate that p75^NTR^‐CTF and p75^NTR^‐FL have opposing functions in melanoma cells, suggesting that the ratio of the two proteins affects the balance between cell death and survival. The presence of distinct p75^NTR^ proteolytic fragments may affect biological outcomes in tumor cells.

## Graphical abstract

The p75^NTR^ carboxyl‐terminal fragment (p75^NTR^‐CTF) promotes melanoma cell proliferation and survival, as well as increasing sorafenib resistance *in vivo* and *in vitro*. Moreover, p75^NTR^‐CTF activates the nuclear factor kappa B pathway and enhances the mRNA and protein levels of its downstream genes. On the contrary, full‐length p75^NTR^ inhibits these processes. Overall, we found that p75^NTR^‐CTF and full‐length p75^NTR^ have opposing functions in melanoma cells.

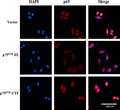

**Abbreviations**
aa: amino acids
ADAM: a disintegrin and metalloproteinase
bFGF: basic fibroblast growth factor
CCK‐8: Cell Counting Kit‐8
cdc2: cyclin‐dependent kinase 1
CDK: cyclin‐dependent kinase
c‐IAP: inhibitor of apoptosis protein
CTF: carboxyl‐terminal fragment
FL: full‐length
FLIP: caspase‐8 inhibitor
ICD: intracellular domain
IκB: inhibitor of κB
IL‐8: interleukin‐8
JNK: Jun N‐terminal kinase
NF‐κB: nuclear factor kappa B
p75^NTR^: p75 neurotrophin receptor
qRT‐PCR: quantitative RT‐PCR
SD: standard deviation
VEGF: vascular endothelial growth factor

The p75 neurotrophin receptor (p75^NTR^, also known as NGFR or CD271), is a 75‐kDa glycosylated transmembrane protein mainly involved in modulating the homeostasis between neural cell survival and death [1, 2]. This p75^NTR^ receptor is composed of extracellular cysteine‐rich repeats that are required for ligand binding, a single‐transmembrane domain and an intracellular death domain [3, 4, 5]. The receptor p75^NTR^ is widely expressed in the central and peripheral nervous system, where it serves as the receptor of multiple neurotrophins [6]. However, p75^NTR^ has been identified in most of the cancer cells and found to be involved in proliferation, survival, apoptosis and migration of tumor cells. For instance, p75^NTR^ inhibits apoptosis and increases cell survival in schwannoma [7] and breast cancer [8, 9]. The overexpression of p75^NTR^ promotes tumor migration and invasion in oral squamous cell carcinoma [10] and glioma [11]. On the contrary, high expression of p75^NTR^ inhibits the nerve growth factor‐stimulated migration or growth of human prostate cancer cells [12], gastric cancer cells [13] and bladder tumor cells [14]. Hence p75^NTR^ performs diverse but sensitized functions according to specific tumor types.

The roles of p75^NTR^ in carcinogenesis have largely been attributed to several signaling pathways, including Ras homolog gene family member A (RhoA), Jun N‐terminal kinase (JNK), mitogen‐activated protein kinase and nuclear factor kappa B (NF‐κB) [15, 16]. Moreover, other than involvement in modulating cellular signaling molecules, p75^NTR^ undergoes sequential cleavages at particular cytoplasmic regions, and these proteolytic fragments may perform disparate functions compared with full‐length p75^NTR^ (p75^NTR^‐FL) [17].

It has been identified that a 15‐amino acid (aa) sequence in the extracellular juxtamembrane of p75^NTR^ is necessary for inducing α‐secretase cleavage, leading to the production of carboxyl‐terminal fragment (p75^NTR^‐CTF) and ectodomain shedding [17, 18]. The members of a disintegrin and metalloproteinase (ADAM) family proteins, especially ADAM‐10 and ADAM‐17, are involved in α‐secretase‐mediated p75^NTR^ cleavage [19, 20]. Subsequently, the transmembrane domain goes through proteolysis via presenilin‐dependent γ‐secretase activity to generate the soluble intracellular domain (ICD) into the cytoplasm [17]. The p75^NTR^‐CTF significantly promotes breast cancer cell survival, whereas ICD remained useless [21]. In contrast, p75^NTR^‐ICD appears to endow cancer cells with resistance to anoikis and promotes metastasis [22]. Furthermore, nuclear translocation of p75^NTR^‐ICD helps modulate transcriptional events [23, 24, 25]. In addition, studies showed that the N terminus of p75^NTR^ can inactivate p53 by direct binding with its central DNA binding domain [26, 27]. However, the knowledge of how p75^NTR^ proteolytic fragments play their roles in tumorigenesis is still largely unknown.

This study aimed to examine the diverse functions of p75^NTR^‐FL and p75^NTR^‐CTF in melanoma A375 cell survival and apoptosis. In addition, inverse effects of p75^NTR^‐FL and p75^NTR^‐CTF on sorafenib resistance shall also be investigated both *in vivo* and *in vitro*. The results provide an explanation for the paradoxical role of p75^NTR^ in tumors.

## Materials and methods

### Cell culture

Human malignant melanoma A375 cells were cultured in DMEM with 10% FBS and maintained at 37 °C in a humidified 5% CO_2_ incubator. The medium was exchanged every 2 days with fresh medium to maintain cell activity.

### Plasmids construction and transfection

p75^NTR^‐FL (aa 1–427) and p75^NTR^‐CTF (aa 231–427) sequences of p75^NTR^ were amplified by PCR from a human cDNA library and cloned into vector pLJM1 (Addgene #19319, Watertown, MA, USA) for expression. Primers are listed in Table S1. These plasmids were respectively cotransfected with psPAX2 envelope and pCMV‐VSV‐G packaging plasmids into prepared HEK‐293T cells using Lipofectamine 2000 (Invitrogen, Carlsbad, California, USA) according to the manufacturer's protocol. The medium contains lentiviral particles that were harvested after 48 h and used to infect target cells. Successful infected cells were selected with 1 mg·mL^−1^ puromycin for 10 days.

### Western blot and immunofluorescence

Melanoma A375 cells were harvested and homogenized in lysis buffer containing phenylmethylsulfonyl fluoride (PMSF). The primary antibodies used were p75^NTR^ (#ab52987; Abcam, Cambridge, UK), NF‐κB p65 (#8242; CST, Danvers, MA, USA), IκBα (#9242; CST, Danvers, MA, USA), Phospho‐IκBα (#2859; CST, Danvers, MA, USA), caspase‐3 (ab214430; Abcam, Cambridge, UK), caspase‐9 (ab202068; Abcam, Cambridge, UK), Bax (ab32503; Abcam, Cambridge, UK), Bcl‐2 (ab32124; Abcam, Cambridge, UK), Lamin B1 (Beyotime, Shanghai, China), inhibitor of apoptosis protein 2 (c‐IAP2; #380798; ZEN BIO, Chengdu, China), bFGF (#381676; ZEN BIO, Chengdu, China) and β‐actin (Sino Biological, Beijing, China). β‐Actin or Lamin B1 expression served as an internal control. For immunofluorescence analysis, cells were incubated with anti‐(human NF‐κB p65) IgG (1 : 100). The primary antibody was detected using a Cy3‐labeled IgG (1 : 100; Beyotime, Shanghai, China), and nuclei were stained with 4',6‐diamidino‐2‐phenylindole dihydrochloride (DAPI). Microscopic analysis was performed using a Nikon E600 microscope (Tokyo, Japan).

### Cell viability assay

For cell proliferation assay, A375 cells were transfected with vector, p75^NTR^‐FL and p75^NTR^‐CTF, respectively, and measured by Cell Counting Kit‐8 (CCK‐8) assay (Beyotime, Shanghai, China) at 24, 48, 72 and 96 h according to the manufacturer's instructions. For drug sensitivity, A375 cells were treated with different concentrations of sorafenib, and the cell viability was measured by CCK‐8 at 24, 48 and 72 h.

### Colony formation assay

Colony formation assays were performed with Crystal Violet Staining Kit (Beyotime, Shanghai, China) according to the protocol.

### Quantitative RT‐PCR analyses

Total RNA was extracted using TRIzol reagent (Takara, Tokyo, Japan). cDNA was synthesized from 2.5 µg total RNA using GoScript Reverse Transcription System (Promega, Madison, WI, USA). The mRNA expression was quantified using SYBR Green Mix (Promega, Madison, WI, USA) according to the manufacturer's protocol. Primers are listed in Table S1.

### Flow cytometry analyses

Treated cells were collected and then fixed in precooled 75% ethanol at −20 °C overnight. The cell‐cycle analysis was performed on a flow cytometer (Influx, BD, San Jose, CA, USA). Cellular apoptosis was analyzed using Apoptosis Analysis Kit (Beyotime, Shanghai, China) according to the manufacturer's protocol on a flow cytometer (Influx, BD, San Jose, CA, USA).

### ELISA

Human interleukin‐8 (IL‐8) and vascular endothelial growth factor (VEGF) ELISA kits (Beyotime, Shanghai, China) were performed on cell culture supernatants according to the manufacturer's protocol. The culture supernatants were assayed at a dilution within the linear range of the IL‐8 or VEGF standards, and the concentration of IL‐8 or VEGF in each sample was determined using the standard curve, as indicated by the kit.

### Mouse xenograft studies

For xenograft implantation, 4‐ to 6‐week‐old male BALB/c‐nu mice were purchased from Chongqing Medical University. All mice were bred and housed under a specific pathogen‐free condition. All animal procedures were performed under the ethical guidelines of Laboratory of Animal Welfare and Ethics Committee of the Chongqing Medical University. A375 cells were harvested from mid‐log phase cultures using trypsin‐EDTA. Cells were then pelleted and resuspended in PBS to a final cell count of 1 × 10^7^ mL^−1^. A volume of 0.2 mL of the cell suspension (2 × 10^6^ cells) was injected subcutaneously in the right flank of each male BALB/c nude mouse. Sorafenib (30 mg·kg^−1^) was treated by gavage daily 4 days after the tumor cell injection [28]. Formation of tumors was examined every 4 days, length and width were measured with calipers, and the tumor volumes were calculated. After 1 month, mice were sacrificed by euthanasia, and tumors were separated from the surrounding tissues.

### Statistical analysis

All values were expressed as mean ± standard deviation (SD), and calculations were carried out using graphpad prism 8 statistical software (San Diego, CA, USA) . Student's *t*‐test was used to estimate the statistical significance of the differences between two groups (*P < 0.05; **P < 0.01; ***P < 0.001).

## Results

### p75^NTR^‐CTF promotes cell proliferation and increases sorafenib resistance

**Fig. 1:**
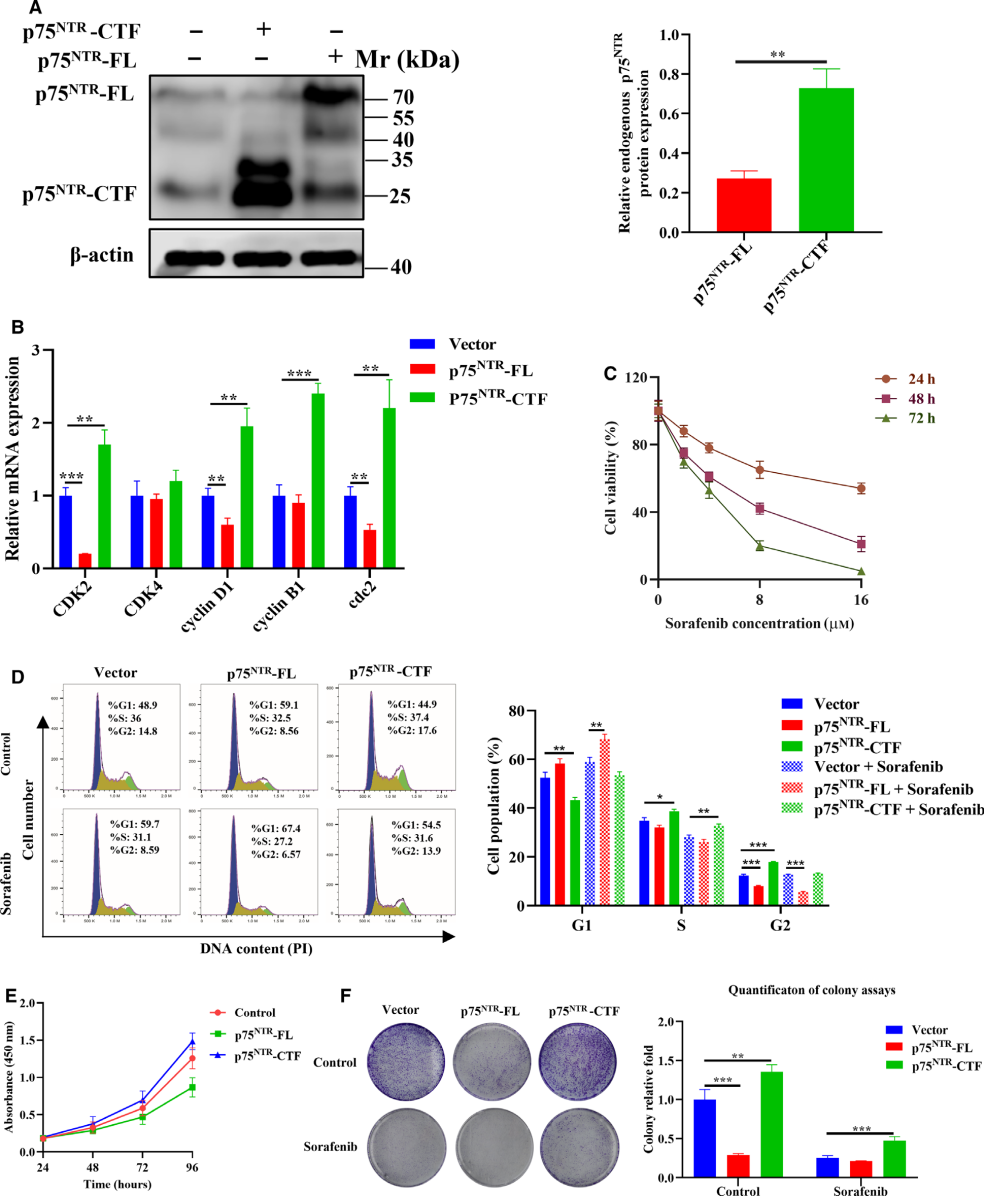
The effects of p75^NTR^‐CTF on cell proliferation and sorafenib resistance. (A) A375 cells were transfected with pCMV‐p75^NTR^‐FL and pCMV‐p75^NTR^‐CTF. Immunoblot results of endogenous and exogenous expressions of p75^NTR^‐FL (75 kDa) and p75^NTR^‐CTF (25 kDa) in A375 cells. (B) Relative mRNA expression levels of cell‐cycle‐related genes (*CDK2*, *CDK4*, *cyclin D1*, *cyclin B1* and *cdc2*) were assessed by qRT‐PCR. (C) A375 cells were treated with sorafenib of different doses (0, 2, 4, 8 and 16 µm) for 24, 48 or 72 h. The effects of sorafenib on A375 cell survival were determined by CCK‐8 assay. (D–F) Stably overexpressed A375 cell lines of p75^NTR^‐FL, p75^NTR^‐CTF and vector were treated with 8 µm sorafenib. Cell cycle was determined by flow cytometry analysis (D), and cell proliferation was determined by CCK‐8 assay (E) and crystal violet staining (F). All values are presented as the mean ± SD from three independent research results; comparison was performed with Student's *t*‐test. *P < 0.05; **P < 0.01; ***P < 0.001.

To detect the proteolysis of p75^NTR^ in melanoma cells, we determined the protein levels of p75^NTR^‐FL/CTF by immunoblotting assay. Results showed that although the bands of p75^NTR^‐FL and p75^NTR^‐CTF were both prominent, the relative intensity of p75^NTR^‐CTF was much higher than p75^NTR^‐FL (Fig. 1A). It is indicated that most p75^NTR^‐FL in A375 melanoma cells proteolytically generated the CTF fragments. To examine the function of p75^NTR^‐FL/CTF in melanoma, we quantified the mRNA levels of cell‐cycle effectors, including cyclin‐dependent kinase 2 (*CDK2*), *CDK4*, *cyclin D1*, *cyclin B1* and cyclin‐dependent kinase 1 (*cdc2*), by quantitative RT‐PCR (qRT‐PCR). Results showed the expression levels of *CDK2*, *cyclin D1* and *cdc2* were significantly reduced in the p75^NTR^‐FL group. Unexpectedly, p75^NTR^‐CTF different from p75^NTR^‐FL drastically increased the levels of *CDK2*, *cyclin D1*, *cyclin B1* and *cdc2* (Fig. 1B). The results hint that overexpression of p75^NTR^‐FL induced cell‐cycle arrest in melanoma cells, which is consistent with previous reports [13]. However, p75^NTR^‐CTF may perform an opposite function to promote cell proliferation.

To confirm this contradictory phenomenon, we constructed stably overexpressed A375 cell lines of p75^NTR^‐FL and p75^NTR^‐CTF. In addition, the cells were then treated with antiproliferative drug sorafenib to detect the effects of p75^NTR^‐FL and p75^NTR^‐CTF on drug resistance. The effective concentration of sorafenib on melanoma A375 cells was determined by CCK‐8, and 8 µm for 48 h was selected for the subsequent experiments (Fig. 1C). By using flow cytometry analyses, it is shown that overexpression of p75^NTR^‐CTF significantly reduced the cell number in the G1 phase, but increased cell numbers in S and G2 phase, respectively, compared with empty vector control and cells overexpressing p75^NTR^‐FL (Fig. 1D). The phenomena were the same with the groups treated with sorafenib, although sorafenib totally decreased cell numbers in S and G2 phase compared with groups not treated with drugs. To reconfirm the results, we used CCK‐8 and crystal violet staining assays to assess cell growth (Fig. 1E,F). Compared with vector and p75^NTR^‐FL cells, the rate of proliferation in A375 cells expressing the p75^NTR^‐CTF was significantly increased when treated with sorafenib. In contrast with p75^NTR^‐FL, p75^NTR^‐CTF promoted cell proliferation and showed increased sorafenib resistance.

### p75^NTR^‐CTF expression inhibits sorafenib‐induced apoptosis *in vivo* and *in vitro*

**Fig. 2:**
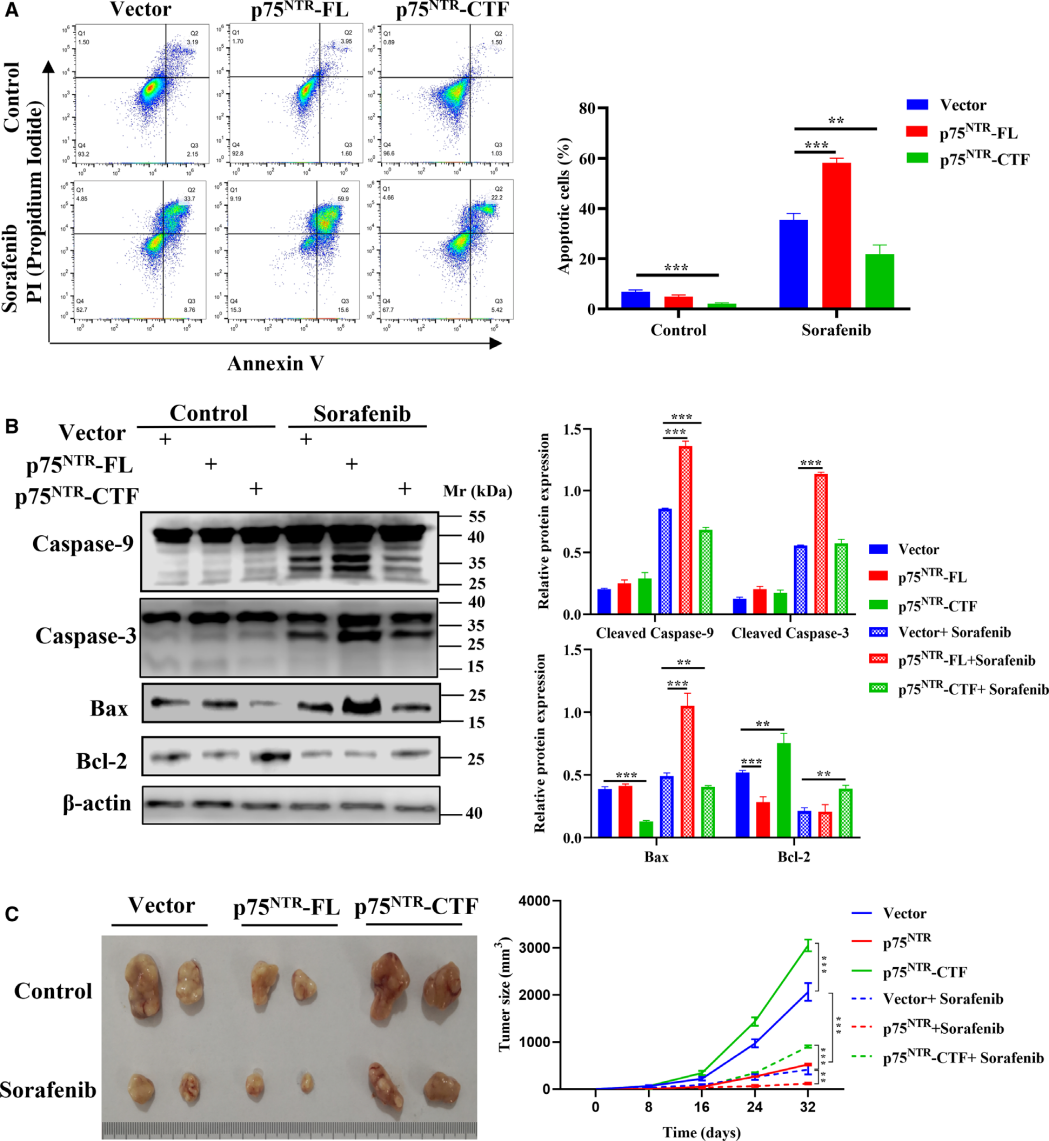
The effects of p75^NTR^‐CTF on sorafenib‐induced cell apoptosis *in vivo* and *in vitro*. (A) Apoptosis was evaluated by flow cytometry after transfection and treatment with sorafenib as indicated. (B) Immunoblot analysis of expression of apoptosis‐associated proteins (caspase‐9, Ccspase‐3, Bax and Bcl‐2) after sorafenib treatment. (C) Nude mouse tumorigenesis test was used to determine cell proliferation and sorafenib resistance of stable expression p75^NTR^‐FL and p75^NTR^‐CTF cell lines. All values shown are mean ± SD of triplicate measurements; comparison was performed with Student's *t*‐test. **P < 0.01; ***P < 0.001.

To further explore the functions of p75^NTR^ to regulate melanoma cell growth, we investigated the effects of p75^NTR^‐FL and p75^NTR^‐CTF on cell apoptosis. The flow cytometry analyses showed overexpression of p75^NTR^‐CTF significantly reduced apoptotic cell number compared with empty vector control (Fig. 2A). As compared with control, overexpression of p75^NTR^‐FL has little effect on cell apoptosis. However, when the cells were treated with sorafenib, p75^NTR^‐FL showed a significant increase in apoptotic ratio, and as expected, p75^NTR^‐CTF exerted the same way to inhibit cell apoptosis. The results were then confirmed by immunoblotting assays (Fig. 2B). Compared with vector, p75^NTR^‐CTF overexpression significantly inhibited caspase‐9 cleavages when the cells were treated with sorafenib. On the contrary, the cleavages of caspase‐3 and caspase‐9 were enhanced in the p75^NTR^‐FL + sorafenib‐treated group compared with vector + sorafenib. In addition, overexpression of p75^NTR^‐CTF significantly reduced the protein levels of apoptosis inducer Bax, while increasing levels of antiapoptotic Bcl‐2, respectively, compared with empty vector control alone or with sorafenib treatment. Nevertheless, p75^NTR^‐FL performed reversed roles to modulate the expression of Bax and Bcl‐2.

To study whether the ability of p75^NTR^‐CTF to inhibit apoptosis leads to enhanced survival of A375 cells *in vivo*, we carried out tumor xenograft assays using p75^NTR^‐FL/CTF stably overexpressed A375 cells. As expected, xenograft tumors derived from p75^NTR^‐FL cells grew remarkably slower and more sensitive to sorafenib than those from control cells in the nude mice (Fig. 2C). While in the presence of sorafenib, tumors derived from p75^NTR^‐CTF cells are much bigger than those derived from the control cells. Notably, there seem to be more visible blood vessels in the tumors derived from p75^NTR^‐CTF cells (Fig. 2C). Thus, these results demonstrate that overexpression of p75^NTR^‐CTF increased the resistance of A375 cells to sorafenib‐induced apoptosis *in vivo*.

### p75^NTR^‐CTF activates the NF‐κB signaling pathway

**Fig. 3:**
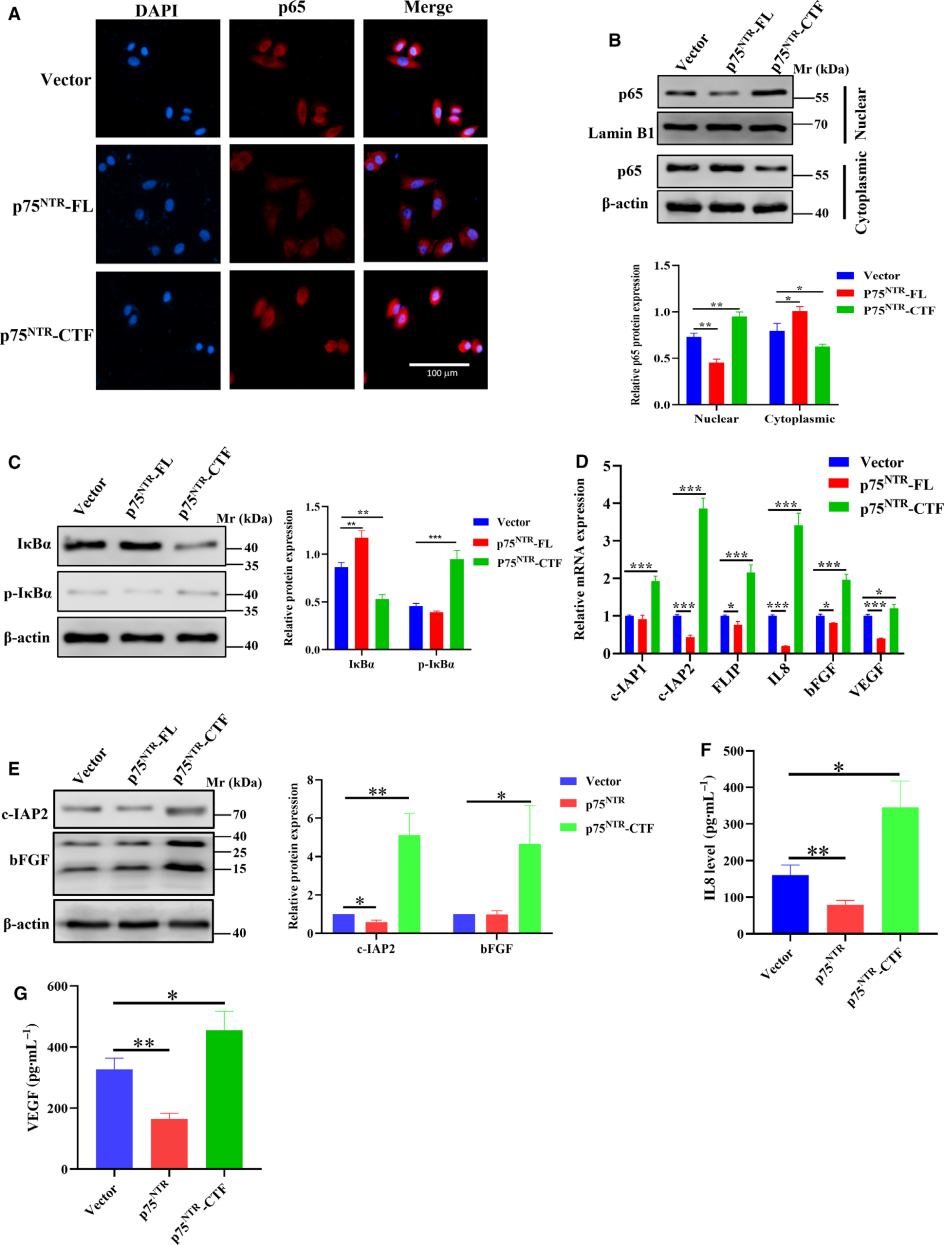
Activation of NF‐κB signaling pathway by p75^NTR^‐CTF. (A) Subcellular localization of NF‐κB p65 (red) was detected by immunofluorescence. Nuclei were stained with DAPI (blue). Scale bar: 100 μm. (B) Cell lysates were extracted from cytosolic and nuclear fractions; the expression levels of p65, β‐actin (cytosolic control) and Lamin B1 (nuclear control) were analyzed by immunoblot analysis. (C) Phosphorylation and total levels of IκBα were assessed by immunoblot analysis. (D) mRNA levels of the antiapoptosis (*c‐IAP1/2*, *FLIP*) and tumor angiogenesis (*IL8*, *VEGF* and *bFGF*) were detected by qRT‐PCR. (E) Protein levels of c‐IAP2 and bFGF were measured by western blot analysis. (F, G) Protein levels of IL‐8 and VEGF in the culture media of the cells were measured by ELISA. Data represent the mean ± SD of three separate experiments. Differences between two groups were compared using Student's *t*‐test. *P < 0.05; **P < 0.01; ***P < 0.001. Scale bar: 100 μm.

Because the NF‐κB signaling pathway is known to stimulate cell proliferation, prevent apoptosis and regulate angiogenesis in several tumors, we sought to investigate the association between the NF‐κB pathway and p75^NTR^ expression. The subcellular locations of the NF‐κB p65 subunit in p75^NTR^‐FL/CTF‐overexpressing cells were first assessed. Fluorescence microscopy showed that the overexpression of p75^NTR^‐CTF leads to migration of the NF‐κB p65 subunit from the cytoplasm to nucleus (Fig. 3A). Meanwhile, immunoblotting assays of cytosolic and nuclear protein extracts also showed that translocation of NF‐κB p65 into the nucleus was enhanced by p75^NTR^‐CTF (Fig. 3B). Inversely, most NF‐κB p65 was observed in the cytoplasm in cells transfected with p75^NTR^‐FL (Fig. 3A,B). NF‐κB p65 as one of the subunits of NF‐κB heterodimerizes with the other subunits p50 or p52, and nuclear localization of the complex is a key to activate the signaling pathway. The results depicted that p75^NTR^‐CTF promoted NF‐κB nuclear accumulation, whereas p75^NTR^‐FL inhibited the process.

NF‐κB activation also results from a disinhibition of inhibitor of κBs (IκBs), and phosphorylation of IκBs will lead the proteins to be degraded by proteasomes [29]. Our results indicated compared with empty vector, the protein levels of IκBα were decreased and phosphorylation of IκBα was significantly increased in p75^NTR^‐CTF‐expressing cells, which is reversed in p75^NTR^‐FL expressing cells (Fig. 3C). In continuation, the mRNA and protein levels of genes activated by NF‐κB were detected by qRT‐PCR, western blot and ELISA assays (Fig. 3D–G). The results showed expression levels of *c‐IAP1/2*, *FLIP* (caspase‐8 inhibitor), *bFGF* (basic fibroblast growth factor), *IL8* (Interleukin‐8) and *VEGF* (vascular endothelial growth factor) were increased in p75^NTR^‐CTF‐expressing cells but decreased in p75^NTR^‐FL‐expressing cells. The results led to the conclusion that p75^NTR^‐CTF activates the NF‐κB signaling pathway, and p75^NTR^‐FL inhibited this process in A375 cells.

## Discussion

The role of p75^NTR^ in tumorigenesis is largely controversial. In breast cancer cells, p75^NTR^ contributes tumor cells to bypass apoptosis via inducing proteome modifications [6]. In addition, siRNA against p75^NTR^ suppresses cell survival and cell migration by decreasing proto‐oncogene tyrosine‐protein kinase Src (SRC) and extracellular‐signal‐regulated kinase (ERK) activation [8, 9]. Although in prostate, gastric and bladder cancers, p75^NTR^ exerts an antitumor effect by inducing cell‐cycle arrest or caspase‐mediated apoptosis [12, 13, 14]. However, the mechanisms underlying the controversial functions of p75^NTR^ in different cancer cells are poorly explained.

This study detected the amount of p75^NTR^‐FL and its segmented p75^NTR^‐CTF in A375 melanoma cells (Fig. 1A). It is demonstrated that p75^NTR^ underwent regulated proteolysis in melanoma cells as described for various different types of cancer cells [17, 18]. Interestingly, the ratio of p75^NTR^‐CTF is higher than that of p75^NTR^‐FL protein, which suggests that p75^NTR^ performs its roles mainly in its proteolytic form in melanoma cells. Furthermore, it is found that p75^NTR^‐CTF exerts tumor oncogenic functions by promoting tumor cell growth in both *in vitro* and *in vivo* models, whereas p75^NTR^‐FL showed entirely opposite functions to inhibit the growth of melanoma A375 cells. Therefore, it is proposed that intracellular setup with distinct presence of p75^NTR^ proteolytic fragments confers the contradictory functions to p75^NTR^ in melanoma A375 cells.

Sorafenib is a multikinase inhibitor and has shown efficacy against numerous tumors in preclinical models, but the chemoresistance is a major obstacle for its use in anticancer therapies [30, 31]. Thus, understanding the molecular mechanisms leading to chemoresistance is a rational step to improve the drug's therapeutic efficacy. A375 cells as malignant melanoma cells are extremely refractory to chemotherapeutic drugs [32]. When the cells were treated with sorafenib, it was found that p75^NTR^‐CTF significantly inhibits caspase‐9 and caspase‐3 cleavages and sorafenib‐induced cell apoptosis, which is a contrasting feature of p75^NTR^‐FL. The results of these findings were reconfirmed by nude mice tumorigenesis assays. Explicitly, p75^NTR^‐CTF enhances sorafenib resistance in melanoma cells and may possibly be a key target in chemoresistance. It is known that ADAM‐17 as a type I transmembrane metalloprotease is involved in the production of p75^NTR^‐CTF [20, 33]. Recently, ZLDI‐8 was reported as a novel ADAM‐17 inhibitor involved in enhanced chemotherapeutic effects of sorafenib in hepatocellular carcinoma cells [34]. It is speculated that ZLDI‐8 would affect proteolysis of p75^NTR^ and inhibit the production of p75^NTR^‐CTF, which requires further in‐depth investigations.

Inflammation as a key trait of cancer cells fosters many hallmarks in anticancer therapy [35, 36]. NF‐κB signaling pathway is one of the major regulators of inflammation in cancer. In tumor cells, activation of NF‐κB contributes to angiogenesis, tumor cell survival, cell proliferation and therapeutic resistance [37, 38]. Herein, our results showed that p75^NTR^‐CTF promotes accumulation of NF‐κB p65 into the nucleus and activates the NF‐κB signaling pathway, whereas p75^NTR^‐FL performs entirely opposite roles to inhibit NF‐κB p65 nuclear accumulation (Fig. 3A,B). Then the study showed that p75^NTR^‐CTF upregulated *c‐IAP1/2* and *FLIP*, which are activated by NF‐κB to confer cells’ antiapoptotic properties. Meanwhile, p75^NTR^‐CTF also stimulates melanoma cells to upregulate *bFGF* expression and secrete IL‐8 and VEGF, which offers an insight into the immunomodulatory functions of p75^NTR^. bFGF and VEGF were reported to drive T cell exhaustion by upregulating the expression of PD‐1, which attenuates the efficacy of anti‐PD‐1 treatment [39, 40]. The results indicated the involvement of p75^NTR^‐CTF in immune therapies of tumor cells. In addition, a recent study also showed that p75^NTR^ high‐expression melanoma fractions are associated with immune exclusion, and p75^NTR^ inhibition restores tumor sensitivity to T cell attack *in vitro* and *in vivo* [41]. Although the development of immune therapies largely prolongs survival rate for patients with late‐stage melanoma, the majority of tumors display either innate or acquired resistance to these therapies. Thus, more targets found will surely increase the therapeutic efficiency. Based on the earlier studies, p75^NTR^‐CTF is predicted to be a novel target for immune therapies, although more investigations are required.

In conclusion, the findings of the study demonstrated that in melanoma A375 cells, p75^NTR^ protein is sensitive to proteolysis to generate p75^NTR^‐CTF, which performs opposite functions compared with p75^NTR^‐FL. p75^NTR^‐CTF promotes cell survival, proliferation and therapeutic resistance by upregulating NF‐κB signaling cascades. In contrast, p75^NTR^‐FL inhibits these processes. It suggested that different proportions of p75^NTR^‐CTF/p75^NTR^‐FL could regulate the balance of cell death and survival. Our findings also indicated that p75^NTR^‐CTF may be a potential target protein for the treatment of patients with melanoma, and inhibitors of p75^NTR^ proteolysis may favor the therapies.

## Conflict of interest

The authors declare no conflict of interest.

## Author contributions

MZ and CB designed and performed all the experiments. MZ drafted the manuscript. YW analyzed the data. FNM gave suggestions to the revision of the manuscript. JG performed animal experiments. All authors approved the final manuscript and agreed to be accountable for all aspects of the work.

## Supporting information


**Table S1.** Primer sequences.

## Data Availability

The data that support the findings of this study can be obtained from the corresponding author on reasonable request.
