# Supplementary material for: The p75NTR and its carboxyl‐terminal fragment exert opposing effects on melanoma cell proliferation and apoptosis via modulation of the NF‐κB pathway
Source: FEBS Open Bio. 2020 Dec 16;11(1):226–36. doi: 10.1002/2211-5463.13047 (PMC7780107; doi:10.1002/2211-5463.13047)
Supplement: Supplementary file 1 — Table S1. Primer sequences. [file FEB4-11-226-s001.docx]

**Supplement Table 1**：Primer sequences

| Name | Forward primer (5’-3’) | Reverse primer (5’-3’) | Length (bp) |
| --- | --- | --- | --- |
| p75NTR-FL | CGAGCTAGCAGTCGAGCGCTGCCG | CGAATTC TCACACCGGGGATGTGGCAGTGGACTCACT | 1328 |
| p75NTR-CTF | ATTGCTAGCATGGGCAGCTCCCAGCCCGTGGTG | CGAATT CTCACACCGGGGATGTGGCAGTGGACTCACT | 595 |
| CDK2 | CCAGGAGTTACTTCTATGCCTGA | TTCATCCAGGGGAGGTACAAC | 90 |
| CDK4 | CTGGTGTTTGAGCATGTAGACC | GATCCTTGATCGTTTCGGCTG | 88 |
| cyclin D1 | GCTGCGAAGTGGAAACCATC | CCTCCTTCTGCACACATTTGAA | 135 |
| cyclin B1 | AATAAGGCGAAGATCAACATGGC | TTTGTTACCAATGTCCCCAAGAG | 111 |
| cdc2 | AAACTACAGGTCAAGTGGTAGCC | TCCTGCATAAGCACATCCTGA | 148 |
| c-IAP1 | AGCACGATCTTGTCAGATTGG | GGCGGGGAAAGTTGAATATGTA | 102 |
| c-IAP2 | TTTCCGTGGCTCTTATTCAAACT | GCACAGTGGTAGGAACTTCTCAT | 96 |
| FLIP | AGGAGCAGGGACAAGTTACAG | GGACAATGGGCATAGGGTGTT | 105 |
| IL8 | ACTGAGAGTGATTGAGAGTGGAC | AACCCTCTGCACCCAGTTTTC | 112 |
| bFGF | AGAAGAGCGACCCTCACATCA | CGGTTAGCACACACTCCTTTG | 82 |
| VEGF  β-actin | AGGGCAGAATCATCACGAAGT  CTCGACACCAGGGCGTTATG | AGGGTCTCGATTGGATGGCA  CCACTCCATGCTCGATAGGAT | 75  114 |

|  |  |  |
| --- | --- | --- |
|  |  |  |
|  |  |  |
|  |  |  |
|  |  |  |
|  |  |  |
|  |  |  |
|  |  |  |
|  |  |  |
|  |  |  |
|  |  |  |
|  |  |  |
|  |  |  |
|  |  |  |
|  |  |  |
|  |  |  |
|  |  |  |
|  |  |  |
|  |  |  |
|  |  |  |
|  |  |  |
|  |  |  |
|  |  |  |
|  |  |  |
